# Maternal Height as an Independent Risk Factor for Neonatal Size Among Adolescent Bengalees in Kolkata, India

**DOI:** 10.4314/ejhs.v20i3.69444

**Published:** 2010-11

**Authors:** Samiran Bisai

**Affiliations:** 1 Department of Anthropology, Vidyasagar University, Midnapore, West Bengal, India; 2 Society for Applied Studies, CF -198, Sector - I, Salt Lake City, Kolkata - 700 064, West Bengal, India.

**Keywords:** India, Bengalee, adolescents, maternal, neonates, Kolkata

## Abstract

**Background:**

Low Birth Weight is a major public health problem in developing countries. The causes of LBW are multifactorial including complication during pregnancy, genetic, environmental, social-cultural, demographic and nutritional variables. Comparison of anthropometric risk factors for neonatal size of adolescent mothers are lacking from West Bengal. Therefore, this study was undertaken to identify maternal anthropometric characteristics, which most strongly influence neonate weight and length among Bengalee mothers.

**Methods:**

A hospital based cross-sectional study was undertaken during 2004 in a Government general hospital in South Kolkata, India. A total of 76 adolescent (age<20years) pregnant women were enrolled from obstetric ward who were admitted for delivery. Due to 4 perinatal deaths; a total of 72 adolescent mother- baby pairs were included in this analysis. Anthropometric measurements were undertaken immediately after delivery following stabilization as well maternal body mass index (BMI) was calculated using standard formula.

**Results:**

The prevalence of low birth weight (LBW) in the present study was 52.8%. The results revealed that 30.6% of mothers were undernourished (BMI<19.8 kg/m^2^). It was noted that about 64% of undernourished mothers delivered LBW baby. Linear regression analyses of neonatal weight and length as dependent variables revealed that in both cases, maternal height had the most significant impact. It showed 12.9% (birth weight) and 16.1% (birth length) of variation. Moreover, the proportion of LBW was 75%, 52.3% and 25% among short (height ≤145 cm), average (146–155cm) and tall (>155cm) mothers (x^2^=6.855, p<0.01), respectively. Short mothers had 2.74 and 9.0 fold greater risk of delivering LBW baby than average and tall mothers. In contrast, mean birth weight and length of baby was lower in short mother than their counterparts.

**Conclusion:**

This study revealed that maternal height had the strongest significant impact on neonate size. This strong association could have serious health implications for Bengalee adolescent mothers. However, since this is a preliminary finding, it needs validation using a larger sample of adolescent mothers.

## Introduction

Low Birth Weight (LBW; birth weight <2500g) (1) is a major public health problem in developing countries (2) particularly in the Indian subcontinent, where the LBW with 30–50% LBW rate, which are among the highest in the world (3). The causes of LBW are multifactorial (4–6), associated with medical complication in pregnancy, genetic, environmental, socio-cultural, demographic and nutritional variables. In addition, other factors related with maternal anthropometry (7) such as maternal weight, height, BMI, arm circumference and head circumference have impact (8).

It was well documented that malnourished mothers are more prone to have LBW babies (4) and pregnancy complications (9). Generally, malnourished girls tend to have short stature in adulthood and they have high rates of adverse pregnancy outcome such as perinatal mortality and prematurity (10). Several studies from India observed very high rates of LBW babies among mothers with height less than140cm (11–13). Another study reported 216g-birth weight variation between short (<143 cm) and tall (>162cm) mothers (6) and Bhatia et al (14) noticed birth weight increased as maternal height increased. Moreover, WHO collaborative study of maternal anthropometry and pregnancy outcome recommends the use of maternal height and weight for screening in its service application (7).

The causes and consequences of LBW in different ethnic groups are of much interest to epidemiologists, health care workers, human biologists and biological anthropologists. However, studies that have compared these risk factor profiles of teenage mothers are lacking from West Bengal. Moreover, to the best of the author's knowledge there exists no published report, which deals with the association of maternal anthropometric characteristics and neonatal size among adolescent Bengalee mothers.

In view of this, this study was undertaken to identify maternal anthropometric characteristics, which most strongly influence neonate weight and length among Bengalee mothers.

## Subjects and Methods

A hospital based cross-sectional study was undertaken during 2004 in a Government general hospital in south Kolkata, India. This hospital serves the needs of patients belonging to the lower - middle class socio-economic group. The estimated minimum sample size (n=72) was calculated using a formula to determine sample size for single proportion population, taking the prevalence (p) of LBW 23% in the same hospital (15) and desired precision of 10% at the 95% confidence interval. All together 76 adolescent (age <20 years) pregnant women were enrolled randomly three days in a week from obstetric ward those who were admitted for delivery. The mothers and baby pairs who were not suffering from any significant medical and surgical disorders at the time of enrollment were included. For cases, birth data was not collected for four cases of perinatal death. Therefore, a total of 72 adolescent mother- baby pairs were included for the present analyses.

Monthly family income was recorded to the nearest year and Rupee (Rs.), respectively. All anthropometric measurements were undertaken immediately (within 24 hours) after delivery following stabilization using standard protocol (16). Mother weight and height was made and recorded to the nearest 100g and 0.1cm, respectively, using Salter digital weighing scale and anthropometer rod. Similarly, head circumference and mid upper arm circumference was measured to the precision of 0.1cm using non-stretchable fibre tape. Biceps and triceps skin fold thickness was measured using Lange skin-fold caliper to the nearest 0.2mm, respectively. Maternal body mass index (BMI) was calculated using standard formula. While, newborn birth weight and length was measured by using triple beam balance and locally made neonatometer to the nearest 1g and 0.1cm, respectively.

Ethical Committee of the Society for Applied Studies, Kolkata, approved the study protocol. A written consent was obtained from each mother. Information on age, ethnicity, years of formal schooling, maternal obstetric, age, family size and monthly income were obtained using a questionnaire.

The distributions of continuous variables were not significantly skewed thus, not necessitating their transformation. Linear regression analyses were used to test for the impact of independent variables. Neonate birth length and weight were used as dependent variables. Odds ratio (OR) and 95% confidence interval (CI) was calculated using standard formula. All statistical analyses were performed using SPSS for windows version 7.5. Statistical significance was considered at p < 0.05.

## Results

The average monthly family income was Rupees 3665.30 (SD ±2166.7). It must be noted here that the exchange rate during the study period was 46 rupees to approximately 1 USD. The mean age and gestational age were 18.3 (0.8) years and 38.1 (2.4) weeks, respectively. The mean family size and formal years of schooling were 4.7 (2.4) person and 5 (3.3) years, respectively.

Anthropometric characteristics of maternal and neonates expressed as means, standard deviation, minimum and maximum values are presented in *Table* 1. The mean (SD) of weight, height, head circumference, MUAC, biceps and triceps skin-fold were 46 (5.9) kg, 149.4 (6) cm, 51.9 (1.5) cm, 22.5 (1.9) cm, 3.7 (1.3) mm and 8.6 (3.2) mm, respectively. The mean (SD) BMI of mothers' was 20.6 (2.2) kg/m^2^. Moreover, Mean birth weight and birth length baby was 2474 (409) gram and 47.3 (2.1) cm, respectively.

**Table 1:** Maternal and newborn anthropometric characteristics, South Kolkata, 2004.

Characteristics	Mean	SD	Minimum	Maximum
**Maternal:**				
Weight (kg)	46.0	5.9	32.3	59.3
Height (cm)	149.4	6.0	128.9	166.4
Head circumference (cm)	51.9	1.5	48.4	56.8
MUAC (cm)	22.5	1.9	18.2	27.1
Biceps (mm)	3.7	1.3	2.0	7.0
Triceps (mm)	8.6	3.2	3.0	16.0
BMI (kg/m^2^)	20.6	2.2	15.6	27.9
**Newborn:**				
Birth weight (g)	2474	409	1281	3441
Birth length (cm)	47.3	2.1	40.7	51.3

**Figure 1:**
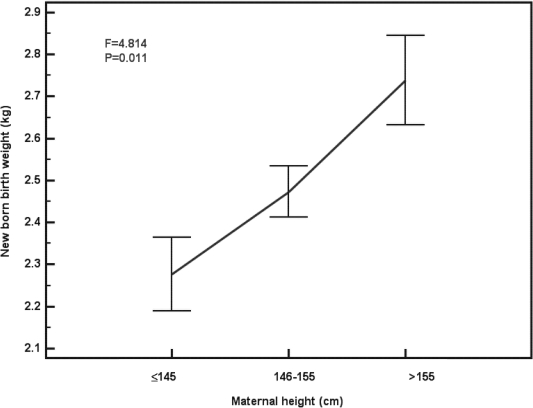
Mean (Se) Birth Weight by Maternal Height, South Kolkata, 2004.

**Figure 2:**
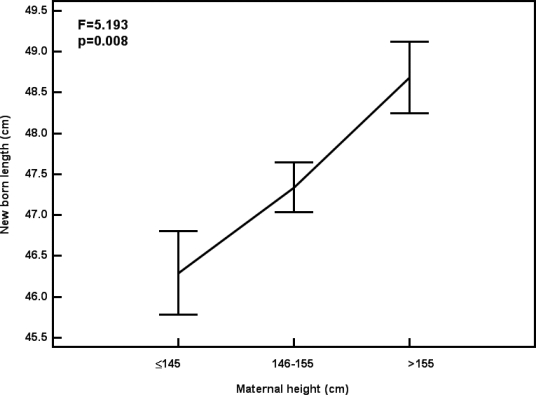
Mean (se) Birth Length by Maternal Height, South Kolkata, 2004.

The prevalence of low birth weight (birth weight<2.5kg) was 52.8%. The results revealed that 30.6% of mothers were undernourished (BMI<19.8 kg/m^2^) and about 64% of undernourished mothers delivered LBW baby. Moreover, the proportion of LBW was 75 %, 52.3% and 25% among short (height ≤145 cm), average (146–155cm) and tall mothers (>155cm). Short mothers had 2.74 (95%CI: 0.67–12.04) and 9 (95% CI: 1.24–78.65) fold greater risk of being LBW baby than average and tall mothers. There was a significantly decreasing trend with advancement of maternal height (x^2^=6.855, p<0.01). In contrast, mean birth weight and length of baby was lower for short mothers, mean birth weight (figure 1) and length of baby (figure 2) was significantly increased with increment of maternal height.

Linear regression analyses of neonate weight and length as dependent variables revealed that in both cases, maternal height had the most significant impact. It explained 12.9% (birth weight) and 16.1% (birth length) of variation. Maternal weight and head circumference also had significant impact on neonate weight and length. It appeared that none of the other maternal anthropometric characteristics had significant effect on the two dependent variables (Table 2)

**Table 2:** Regression analyses of maternal anthropometry on newborn size, South Kolkata, 2004.

Dependent Variables	Independent Variables	B	SeB	Beta	F	P	AdjR^2^
**Birth weight**							
	Weight	0.02299	0.008	0.335	8.886	0.004	10.0 %
	Height	0.05590	0.008	0.375	11.480	0.001	12.9 %
	Head cir.	0.07345	0.032	0.261	5.118	0.027	5.5 %
	MUAC	0.02763	0.025	0.133	1.255	0.266	0.4 %
	Biceps	0.06345	0.037	0.202	2.969	0.089	2.7 %
	Triceps	0.00574	0.015	0.044	0.138	0.711	−1.2 %
	BMI	0.02290	0.022	0.126	1.127	0.292	0.2 %
**Birth length**							
	Weight	0.09724	0.039	0.283	6.077	0.016	6.7 %
	Height	0.14200	0.037	0.415	14.576	0.000	16.1 %
	Head cir.	0.35200	0.163	0.249	4.626	0.035	4.9 %
	MUAC	0.05756	0.125	0.055	0.213	0.646	−1.1 %
	Biceps	0.23700	0.187	0.150	1.621	0.207	0.9 %
	Triceps	0.01932	0.077	0.030	0.062	0.804	−1.3 %
	BMI	0.03352	0.109	0.037	0.094	0.760	−1.3 %

## Discussion

The mean maternal height and weight in this study were similar to those reported by the WHO (7) collaborative study conducted in Pune and Hyderabad, India. The mean maternal head circumference in the present study was similar to that reported in a recent study from Pune, India (17).

Most importantly, this study provided strong evidence that maternal height had the strongest significant impact on newborn size (birth weight and birth length). It is well established that socio-economic status and ethnicity influences height. Stunting is a consequence of long-term poor nutritional intake and is the best indicator of decreased growth in children over an extended period. Stunting has been associated with poorer cognition and school achievement in later childhood (18). Stunting has also been linked to the perpetuation of the cycle of undernutrition by causing low birth weight among offspring of the stunted mother (19). The height of the mother is a well-known predictive index of perinatal mortality and morbidity. A study from India reported high incidence of LBW (29.% vs 24.2%) infants in mothers with height less than145cm than the mothers with greater than 145cm (5). They had 1.32 times risk of giving birth to LBW in comparison to mothers > 145cm in height. Several other studies have reported that short stature mother had greater risk for adverse pregnancy outcome (11, 14, 20).

Thus, this study clearly indicated the strong influence of maternal height on neonate size among adolescent Bengalee mothers. Maternal height alone accounted for 12.9% and 16.1% variation in neonate weight and length, respectively. This strong association could have serious health implications among adolescent mothers in the developing country where most of them suffer form prolonged nutritional deficiency. However, since this is a preliminary finding, it needs validation using a larger sample of adolescent Bengalee mothers. If validated, an appropriate maternal height cut-off point for neonate size could be derived. Based on this cut-off point, high-risk adolescent pregnancies can be identified for appropriate nutritional intervention.
